# Preserved tactile distance estimation despite body representation distortions in individuals with fibromyalgia

**DOI:** 10.3389/fpain.2024.1414927

**Published:** 2024-07-25

**Authors:** Tania Augière, Morgane Metral, Martin Simoneau, Catherine Mercier

**Affiliations:** ^1^ Center for Interdisciplinary Research in Rehabilitation and Social Integration (Cirris), Quebec, QC, Canada; ^2^ School of Rehabilitation Sciences, Faculty of Medicine, Laval University, Quebec, QC, Canada; ^3^ Univ. Savoie Mont Blanc, Univ. Grenoble Alpes, LIP/PC2S, Grenoble, France; ^4^ Department of Kinesiology, Faculty of Medicine, Laval University, Quebec, QC, Canada

**Keywords:** chronic pain, body schema, body image, multisensory integration, tactile perception, sensory weighting

## Abstract

Our mental representation of our body depends on integrating various sensory modalities, such as tactile information. In tactile distance estimation (TDE) tasks, participants must estimate the distance between two tactile tips applied to their skin. This measure of tactile perception has been linked to body representation assessments. Studies in individuals with fibromyalgia (FM), a chronic widespread pain syndrome, suggest the presence of body representation distortions and tactile alterations, but TDE has never been examined in this population. Twenty participants with FM and 24 pain-free controls performed a TDE task on three Body regions (upper limb, trunk, lower limb), in which they manually estimated the interstimuli distance on a tablet. TDE error, the absolute difference between the estimation and the interstimuli distance, was not different between the Groups, on any Body region. Drawings of their body as they felt it revealed clear and frequent distortions of body representation in the group with FM, compared to negligible perturbations in controls. This contrast between distorted body drawings and unaltered TDE suggests a preserved integration of tactile information but an altered integration of this information with other sensory modalities to generate a precise and accurate body representation. Future research should investigate the relative contribution of each sensory information and prior knowledge about the body in body representation in individuals with FM to shed light on the observed distortions.

## 1 Introduction

Body representation can be defined as the mental representation of our body (i.e., its size, shape, weight, posture, etc.). It is commonly divided into two distinct but complementary representations: the body image and the body schema. The body image represents the perceptual and emotional representation of our body (1). This conscious representation is involved in tasks such as the subjective evaluation of our body, the denomination of body parts, or the drawing of our figure. The body schema, on the other hand, reflects the sensorimotor representation essential to plan and control actions (2, 3). This unconscious representation is involved in mentally rotating a limb, determining if we are tall enough to reach an object, or flexible enough to perform a yoga pose. Both representations rely on the integration of sensory afferents, such as visual, proprioceptive, and tactile information (2, 4–6).

Several authors have demonstrated the critical importance of tactile information for body representation by manipulating tactile afferents (5, 7–9). For example, removing or increasing tactile afferent information (using anesthesia or electrical stimulation, respectively) has been shown to alter the perceived size of a given body part (8). Disrupting the congruency between tactile and visual information can also alter body representation, provoking a body illusion. In full-body illusions, participants typically see the body of an avatar in place of their body, via virtual reality, and the illusion is generated by showing the avatar being stroked while the unseen equivalent body part of the participant is also being stroked (10). The integration of the tactile information, congruent with the visual information distorts the body representation of the participants and leads to a feeling of ownership over the avatar (5, 10–12). Studies show that embodiment of an overweight (11) or older (12) avatar was associated with changes in the participants’ perception of their body size and abilities. Therefore, a precise and accurate perception of tactile information is essential to produce an accurate body representation.

Tactile distance estimation (TDE) tasks are often used to assess tactile perception. In this task, a pair of tactile stimuli is applied to the body, and participants must estimate the distance between these stimuli without vision. The estimation can be relative (comparison to the distance between another pair of tactile stimuli) or absolute (verbal estimation in millimeters or manual estimation by showing the distance between the index and thumb). An underestimation or overestimation of the distance is sometimes interpreted as an underestimation or overestimation (respectively) of the size of the stimulated body site. Indeed, studies suggest that perception of tactile distance on the skin cannot be immediately encoded at the first level of somatosensory processing, as can be the pressure of a tactile stimulus for example (13, 14). Tactile perception may require the mediation of a mental representation of the body, in the higher somatosensory areas, representing physical properties of the body between the two stimulated skin regions (13, 14).

Several studies focused on the link between TDE and body representation. One of them measured the effect of a body resizing illusion on TDE, using a paradigm derived from the Pinocchio illusion (15). Participants held their left index finger with their right arm while a vibration was applied to their right biceps (16). The vibration created an illusion of elongation of the left index finger (16). During the illusion, the participants were asked to compare a tactile distance applied on the elongated finger and the same distance applied on the forehead [relative judgment; (16)]. Results show that they were more likely to perceive the distance applied on the finger as longer than a condition with no illusion. This suggests a link between the body representation and the TDE. Even momentary changes in body representation, such as changes in posture, can impact TDE. Longo and colleagues showed that TDE on the hand was worse when the fingers were pulled apart than when they were together (17). Finally, patients with anorexia nervosa perceived distances over the abdomen and thigh, emotionally biased body parts, as significantly wider than controls, but not when it was given in the vertical orientation (18). The authors did not find any difference between horizontal and vertical orientations over the sternum, used as a neutral body part. The distortion of the patients’ body image, which concerns the dimension of width, would impact the body representation and the TDE task.

Body representation seems to be altered in individuals with chronic pain. Although assessment of such alterations is subjective by nature, distortions have been reported consistently in independent studies conducted in various countries and include feelings of variations in the size (5, 19–26), weight (21, 22), temperature (19), or ownership (19, 21, 24) over the painful body parts, as well as a hypersensitivity to body illusions (27–29). These alterations can be disturbing for patients and are often accompanied by feelings of distress (21, 30–34). Some studies show these alterations are more present in individuals with worse tactile perception (23, 35, 36).

However, the only two studies measuring tactile perception with the TDE task in participants with chronic pain show conflicting findings. Adamczyk and colleagues assessed the TDE in participants with low back pain and showed an association between a high pain intensity and a larger distance underestimation on the lower back (37). On the contrary, Reinersmann et al. found no link between clinical variables and the estimation error in patients with complex region pain syndrome (CRPS). They also found that TDE did not differ between the participants with CRPS and pain-free participants (CTRL group). Interestingly, however, when participants were required to select the image that best matched the size of their painful (or dominant, for the CTRL group) hand, the CRPS group was more likely to choose a bigger hand than the CTRL group (38). This result points to distortions of the representation of the hand despite the absence of alteration of the TDE on the hand in individuals with CRPS.

Fibromyalgia (FM) is a chronic pain syndrome that affects 2%–3% of the population (39, 40). An important characteristic of fibromyalgia (compared to low back pain and CRPS in which previous studies have been conducted) is that the pain is widespread, which might have a different impact on body perception compared to a more localized type of pain. To our knowledge, TDE has never been assessed in individuals with FM. Given the importance of tactile information for body representation and the tactile alterations (28, 41–43) and body representation distortions (20, 21, 24, 28, 32, 33, 44, 45) reported in FM, it seems relevant to compare TDE and body representation in participants with FM and pain-free controls.

The main goal of this case-control study was to compare the TDE and the body representation of individuals with FM and pain-free individuals (CTRL). Objective 1 was to compare the TDE errors between participants with FM relative and pain-free participants. We expected the FM group to make more significant errors than the CTRL group. Objective 2 was to explore TDE errors according to the distribution of pain in individuals with FM. We anticipated errors to be larger on more painful body regions than on less painful body regions. Objective 3 was to describe the body representation between the groups. The hypothesis was that FM participants would show more body representation distortions compared to pain-free controls.

## 2 Materials and methods

### 2.1 Participants and ethical statement

Twenty participants with FM were recruited via Laval University, the Fibromyalgia Association of Quebec City, the Quebec Research Pain Network, and the Fibromyalgia Association of Montreal. Twenty-four healthy controls, matched to the FM group for age and sex, were recruited via Laval University, the FADOQ Network (a group of organizations for residents of Quebec who are ≥55 years old), and Facebook. The sample size was based on pragmatic considerations on how many participants with FM would be willing to participate, given the fatigue, pain, and mindfog associated with this syndrome. This is in line with the sample sizes of similar studies (20, 38). The inclusion criteria that were common to all the participants were: (1) being ≥18 years old; (2) having normal or corrected-to-normal vision; (3) having no non-neurological sensitive alterations (burns, bruises, etc.) on the non-dominant shoulder, forearm, thigh, leg, and on the back of the neck and lower back; (4) having no neurological disorders; (5) not having undergone surgery in the last three months. Additionally, participants with FM had to have received a diagnosis of FM according to the American College of Rheumatology by a qualified doctor (39, 46, 47). Control participants were excluded if they had a history of chronic pain and/or of acute pain severe enough to interfere with daily functioning in the last month or of acute pain on the day of the participation.

All participants provided their written informed consent before they participated in this study. The experiment was performed in accordance with the Declaration of Helsinki, and the study protocol was approved by the local ethical review board (CIUSSS de la Capitale-Nationale, Quebec City, Canada, #2022-2334 RIS).

### 2.2 Study design

Participants took part in an experimental session of one hour for the CTRL participants and one and a half hours for the FM participants at the Center for Interdisciplinary Research in Rehabilitation and Social Integration (Cirris). Participants filled out questionnaires aiming to determine their handedness, their level of physical activity and, for the FM participants, their clinical characteristics (intensity of pain, location of pain, medication etc.). Then, they performed the TDE task designed to measure the perception of the distance between two tactile stimuli***.*** Finally, to assess body representation, participants completed drawings of their body as they felt it.

### 2.3 Questionnaires

Handedness: The French version of the Edinburgh handedness inventory (48–50) was used to determine manual preference. This information was used to determine which hemibody was tested in the TDE task and was not further investigated.

Physical activity: Physical activity has been associated with body representation (51–54), with physical activity interventions showing a positive effect on body image (54). Therefore, all participants completed the French version of the International Physical Activity Questionnaire [IPAQ; (55–57)] to assess their physical activity intensity in the last seven days. IPAQ's continuous score was calculated for group comparisons (57). Participant's physical activity levels were also described with IPAQ's categorical score (high, moderate, low).

Pain and pain interference: Participants with FM were questioned about their medical history and asked to fill out the French version of the Brief Pain Inventory [BPI; (58)] to assess the severity of their pain and its impact on daily function. Before and after the tactile perception task, participants with FM were asked to rate their pain intensity on a visual analog scale (0–10) on each Body site tested in the task.

### 2.4 TDE task

**Figure 1:**
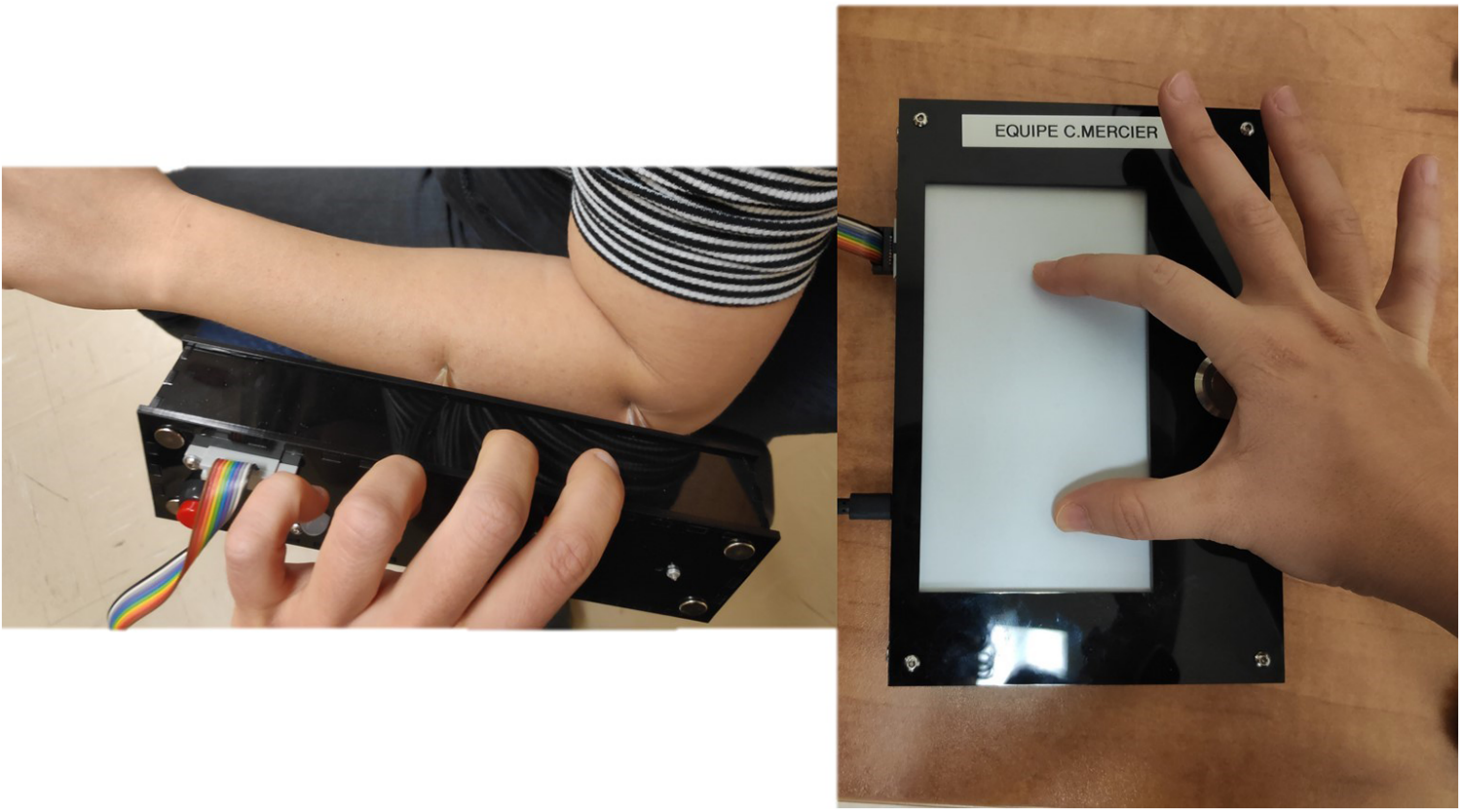
The caliper (left panel) and the touch pad (right panel) used for the TDE task.

Participants were asked to wear clothes that permitted easy access to the skin of the shoulders, the back of the neck, the lower back, the thighs, and the calves, such as a t-shirt with a wide neckline and a loose short or skirt. Participants sat on a comfortable chair in front of a table. An automated and motorized caliper (shown in the left panel of Figure 1) was applied vertically for two seconds on different sites of their non-dominant hemibody. After the application, participants were asked to estimate the distance with the fingers of their opposite (dominant) hand on a touchpad positioned in front of them (shown in the right panel of Figure 1). The caliper and the touchpad were connected via a USB cable to a computer running a web application (SERVO; www.servo.psycho-usmb.fr). The connected application automatically adjusted the distance between the two polyvinyl rods of the motorized part of the caliper (inter-stimuli distance). The application determined the inter-stimuli distance, indicated the body area to be stimulated, and calculated the difference between the participant's estimation and the inter-stimuli distance (in millimeters). Participants had their eyes closed during the caliper application but were allowed to open their eyes while estimating the distance on the tablet. When satisfied with their estimation, they pressed a button to validate their response, and another trial began. If a participant felt only one point of contact, the caliper was applied up to two more times with more pressure, and if the participant still did not feel the two points of contact, the trial was removed from the analysis. A trial was defined as the application of the caliper followed by the estimation by the participant. Three inter-stimuli distances were tested: 50, 65, or 80 mm (participants were unaware of this information). Six Body sites were tested: the dorsal neck (about 2 cm lateral to the C7 vertebra), the lower back (about 2 cm lateral to the L4 vertebra), the shoulder (about 2 cm distal to the greater tubercle of the humerus), the elbow (about 2 cm distal to the lateral epicondyle), the thigh (mid-distant to the hip and the knee, on the lateral side) and the knee (about 2 cm distal to the lateral condyle).

The order of the three inter-stimuli distances and the six Body sites was semi-randomized: no site or applied distance can be repeated twice in a row to prevent participants from getting used to the distance and/or the targeted site. The TDE task was divided into two blocks of 36 trials [(3 inter-stimuli distances × 6 Body sites) × 2 trials], with a break in between. The 6 Body sites were pooled into 3 Body regions: upper limb (UL; shoulder and elbow), trunk (neck and low back), and lower limb (LL; thigh and knee) for further analyses, leading to a total of 12 trials by region. A familiarisation phase was performed on the dorsal non-dominant hand so the participants would be familiar with the task. The familiarisation phase ended when the participants were comfortable with the task, usually after one or two trials.

For each inter-stimulus distance, the estimation error was defined as the absolute difference between the distance estimated by the participants and the distance applied with the caliper. The estimation errors were then averaged across the 3 inter-stimulus distances to obtain a general estimation error for the participant on a given Body region. We chose the absolute difference instead of the signed difference to avoid the cancellation of underestimation and overestimation when calculating the median for each group. The larger this difference is, the larger the error (in either direction) is. The distance estimation was also calculated for each Body region and participant to ensure the participants’ estimations varied according to the inter-stimulus distances.

### 2.5 Body drawings

Participants were instructed to draw the outline of their body as they felt it (as opposed to how they saw it). For example, if a part of their body felt big but visually was not looking big, they should draw it as bigger than its actual size. Participants were told to draw body parts as bigger, smaller, distorted, invisible (if they did not feel it), or any perception they felt. To assist participants, a template of an individual of their gender was provided with discontinued lines on the outline of the body. Participants were allowed to draw on the outline of the template if necessary.

### 2.6 Statistics

Comparisons were considered statistically significant for a *p *< 0.05.

Clinical and demographic data: Clinical and demographic data were synthesized with descriptive statistics, except for the IPAQ continuous score, which was compared between groups with Mann-Whitney U because of a violation of normality (revealed by a Shapiro-Wilk test).

VAS: Since none of the data followed a normal distribution (as shown by a significant Shapiro-Wilk test), and transformations did not resolve the skewness of the data, nonparametric tests were used here too. The differences in pain intensity according to the pooled Body regions in individuals with FM were tested with a Friedman test with the within-subject factor regions (trunk, UL, LL). If a significant result was found, post-hoc comparisons with a Bonferroni correction were performed.

TDE: Since none of the data followed a normal distribution (as shown by a significant Shapiro-Wilk test), and transformations did not resolve the skewness of the data, nonparametric tests were used. NparLD, a non-parametric equivalent of a repeated measures ANOVA (59) was performed on the estimation error, with the within-subject factor Body region (trunk, UL, LL) and the between-subject factor Group (FM, CTRL). NparLD is a robust method for mixed designs with inequivalent samples and does not require normality of distributions and homoscedasticity (59). The statistical tests were performed with IBM SPSS (version 29), except for the nparLD, which was conducted with Rstudio Team (2023). Note that because non-parametric statistics were employed, the descriptive statistics reported include median and interquartile range (IQR).

Body drawings: Drawings were visually assessed by two independent reviewers to determine if body representation distortions were present or not in each of the 6 tested Body sites for each participant. One of the two reviewer was unaware of the hypotheses and group repartition of the participants. The interrater agreement was calculated for each site with Gwet's coefficient (60, 61). The agreement was interpreted as poor (inferior to 0.0), slight (0.0–0.20), fair (0.21–0.40), moderate (0.41–0.60), substantial (0.61–0.80), or almost perfect (0.81–1.00) (61). A consensual decision resolved any discrepancy between the evaluation of the two reviewers.

## 3 Results

### 3.1 Clinical and demographic data

Twenty participants with FM (19 females, 1 male) and 24 pain-free controls (23 females, 1 male) were recruited. The groups were of similar age (FM: median = 44.0, IQR = 15.0 years old; CTRL: median = 42.5, IQR = 30.8 years old). The clinical profiles of the FM group are described in Table 1. For this group, the BPI scores indicated a mean pain severity of 5.4 ± 1.6 /10 and a mean pain interference with daily living of 4.3 ± 2.0 /10. One participant with FM was removed from the analysis for the IPAQ scores because of a lack of answers. Mann-Whitney U indicated no group differences in physical activity level (FM: median = 3,440 MET-min/week, IQR = 4,647; CTRL: median = 2,381 MET-min/week, IQR = 1,941, *p *= 0.334). Twenty-one participants could be considered highly active (11 FM, 10 CTRL), 15 had moderate physical activity (5 FM, 10 CTRL), and 8 (4 FM, 4 CTRL) had low physical activity.

**Table 1:** Clinical profiles of the participants with FM. F, female; M, male; BPI, brief pain inventory; IQR, interquartile range.

Participant	Sex	Age (years)	Currently working?	Pain duration (years)	BPI: pain severity	BPI: Pain interference	Current comorbidities	Pharmacological treatments	Non-pharmacological treatments
S01	F	25	Yes	11	6.0	2.7	Attention deficit disorder, asthma, hypothyroidism, irritable bowel syndrome, migraines	Lyrica	
S02	F	35	Yes	23	4.0	5.0	Arthritis, post-traumatic stress disorder, migraines, occipital neuralgia, morbid obesity	Acetaminophen, lyrica, effexor, cyclobenzaprine, voltaren	
S03	F	53	Yes	48	5.3	4.3	Post-traumatic stress disorder, generalized anxiety disorder, asthma, ulcerative colitis	Cymbalta, lyrica	Physiotherapy, psychotherapy
S04	F	47	Yes	33	7.0	3.3	Irritable bowel syndrome	Amitriptyline, pregabalin, acetaminophen, naproxen	Physiotherapy
S05	F	40	Yes	40	1.8	2.7		Robax-platinum, acetaminophen	Chiropractic, massotherapy, osteopathy
S12	F	31	Yes	8	6.3	4.7	Rheumatoid arthritis, Crohn's disease, endometriosis	Lyrica, acetaminophen	
S13	F	58	No	13	1.8	0.3	Hashimoto's thyroidism, celiac disease	Fluoxetin, naproxen	Osteopathy
S14	F	47	No	32	5.3	3.3		Tramadol, duloxetine, naproxen	Massotherapy, physiotherapy
S15	F	56	Yes	30	4.3	2.4	Hypothyroidism	Pregabalin, duloxetin	Chiropractic, physiotherapy, osteopathy, massotherapy
S16	F	35	Yes	2	4.5	3.1			Massotherapy
S17	F	43	Yes	44	5.8	5.3	Chronic migraines, rheumatoid arthritis, irritable bowel syndrome, generalized anxiety disorder	Lyrica, citalopram, mirtazaprine, acetaminophen	Psychotherapy, chiropractic, osteopathy
S18	F	21	Yes	7	5.5	4.3	Attention deficit and hyperactivity disorder, irritable bowel syndrome, borderline personality disorder, generalized anxiety disorder, triple X syndrome, chronic fatigue syndrome	Duloxetine	Psychotherapy
S22	F	45	Yes	38	5.0	5.7	Generalized anxiety disorder, irritable bowel syndrome	Citalopram, THC oil	Chiropractic
S23	M	66	No	20	7.0	3.3		Duloxetine	Massotherapy
S24	F	39	Yes	5	6.0	5.4	Irritable bowel syndrome, restless leg syndrome, overactive bladder, chronic urticaria	Copaiba essential oil	Massotherapy, osteopathy
S25	F	43	Yes	10	5.3	4.4	Arthritis, migraines	Ibuprofen, acetaminophen, robaxacet	
S34	F	57	No	25	6.5	5.0	Meniere's disease (deafness of right ear), overactive bladder, severe allergies	Cymbalta, naproxen, amitryptyline	Osteopathy, chiropractic, zootherapy
S36	F	53	Yes	8	5.8	6.1	Hypothyroidism, irritable bowel syndrome, sinus arrhythmia, gastric reflux	Cymbalta, naproxen	Psychotherapy, massotherapy, reiki
S37	F	42	Yes	33	5.8	8.7	Depression and suicidal thoughts	Voltaren, naproxen, cannabis	
S43	F	48	Yes	2	2.8	2.4	Hypothyroidism, asthma, sleep apnea	Amitriptyline, duloxetine, cyclobenzaprine	Massotherapy, osteopathy, acupuncture, self-hypnosis
**Median ± IQR**				**21.5 ± 25.0**	**5.4 ± 1.6**	**4.3 ± 2.0**			

The bold values are the group data.

### 3.2 VAS

**Figure 2:**
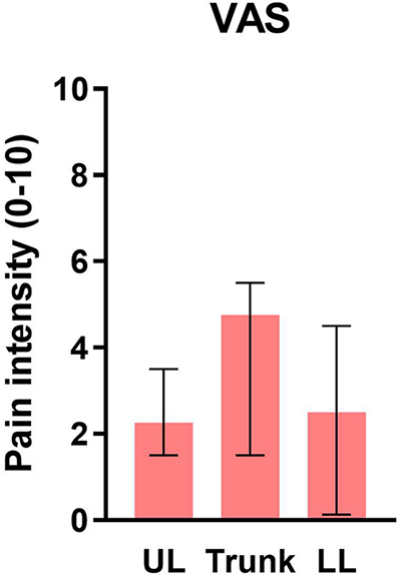
Median pain intensity of participants with FM, over the UL, the trunk, and the LL. Error bars represent the IQR. * indicates a *p* < 0.05; other differences are not signiﬁcant. UL, upper limb; LL, lower limb.

For the pain intensity in the tested zones, the Friedman test revealed a statistically significant effect of region on the pain intensity [*χ*^2^(2)*** = ***9.658, *p* = 0.008]. Post-hoc comparisons showed a greater pain intensity at the trunk than at the UL [*χ*^2^(2)*** ***= 0.875, *p* = 0.017] or the LL [*χ*^2^(2)*** ***= −0.775, *p* = 0.043]. Results are shown in Figure 2.

### 3.3 TDE

Ten trials were excluded (in a total of 4 trials × 3 inter-stimuli Distances × 6 Body sites × 44 participants = 3,168 trials, which accounts for 0.3%) because the participants (nine trials for S24, a FM participant, and one trial for S30, a CTRL participant) did not feel the two tips after three applications.

**Figure 3:**
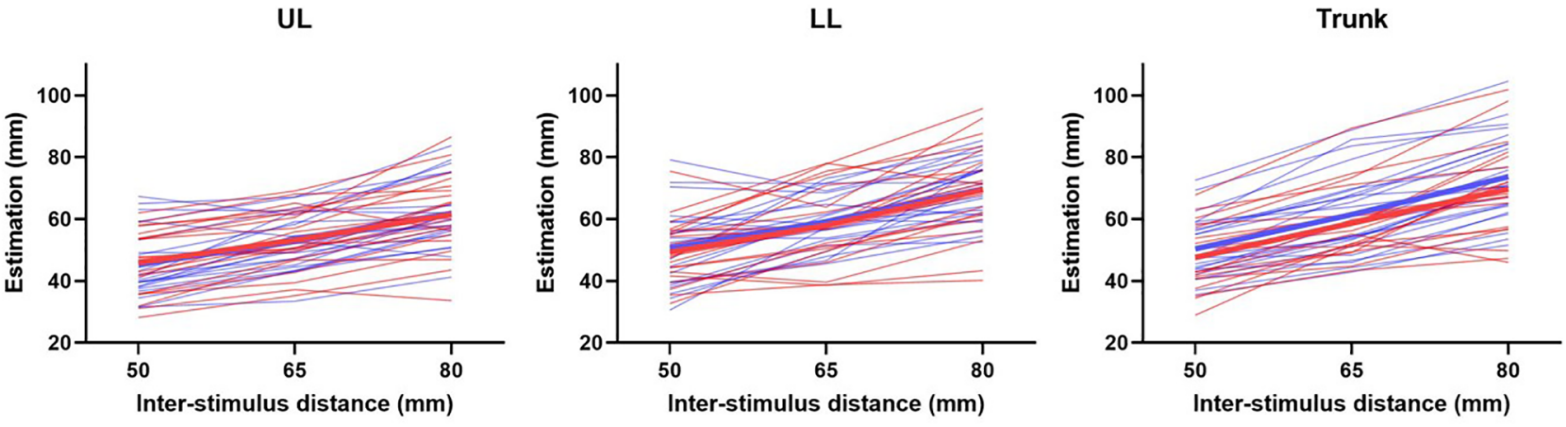
Estimation according to the inter-stimulus distance, for the UL (left panel), the LL (middle panel), and the trunk (right panel). Each thin line represents a participant, of the FM group (in red) or of the CTRL group (blue). The thick line represents the mean for each group. UL, upper limb; LL, lower limb.

The signed difference between the estimated distance and the actual distance for each group and Body region is presented in Figure 3. Substantial interindividual variability was present for all three Body regions and both groups, with both underestimation and overestimation detected in each group. This observation confirms the use of the absolute difference for further analysis. Despite this variability, an apparent augmentation of the estimation according to the actual inter-stimuli distance was observed, which reflects an adequate discrimination of the tactile stimuli for both groups.

**Figure 4:**
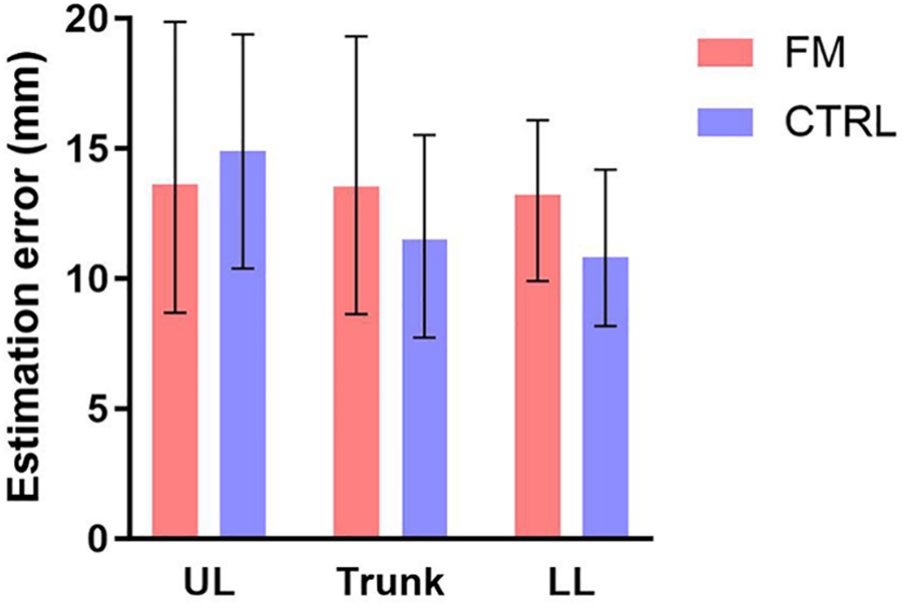
Median error of the estimation of the distance in participants with FM (in red) and CTRL participants (in blue), over the UL, the trunk, and the LL. The error bars represent the IQR. No significant differences were found. FM, participants with fibromyalgia; CTRL, control participants; UL, upper limb; LL, lower limb.

Contrary to our hypothesis, the nparLD showed no effect of the Group [F(1,43) = 1.01, *p* = 0.32] for the estimation error. No effect of the Body region [F(1,43) = 1.44, *p* = 0.24] and no interaction [F(1,43) = 1.54, *p *= 0.22] were found. Results are presented in Figure 4.

### 3.4 Body drawings

**Figure 5:**
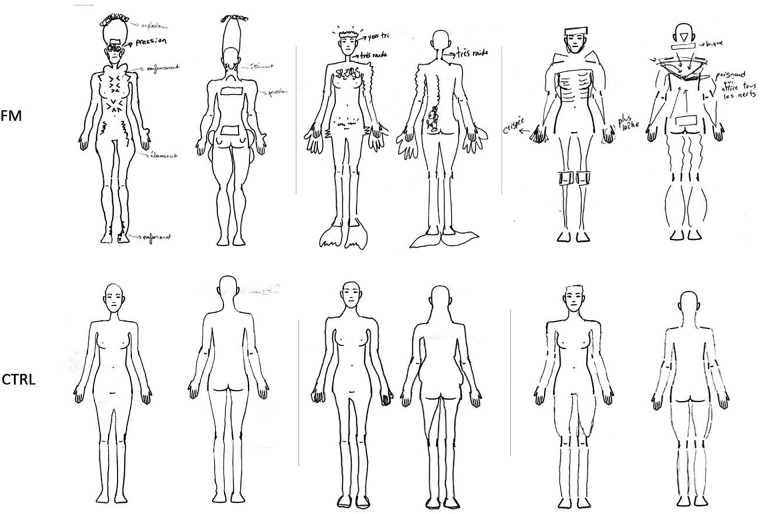
Drawings of 3 representative participants with FM et 3 representative CTRL participants. The participants of the FM group are (from left to right): S17, S12, S34; the CTRL participants are (from left to right): S08, S11, S21. The annotations for S17 indicate an “explosion” of the head, “pressure” on the eyes and shoulders, a “depression” of the thoracic cage, abdomen, and ankles, “shooting pain” in the thighs, and “stretching” of the neck. For S12, annotations demonstrate a sensation of having “very small eyes” and a “very stiff” neck. S34's annotations express a “tense” hand and another “looser” hand, a sensation of having a “brick” behind the head, and of having “a knife pulling on all the nerves” in the middle of the back. FM, participants with fibromyalgia; CTRL, control participants.

The drawings of 6 representative participants (3 participants with FM and 3 controls) are depicted in Figure 5; the drawings of all participants are available in the Supplementary material. Interrater agreement ranged from substantial to near perfect (gwetscore_shoulder_ = 0.91, gwetscore_elbow_ = 0.75, gwetscore_thigh_ = 0.78, gwetscore_knee_ = 0.83, gwetscore_neck_ = 0.91, gwetscore_lower back_ = 0.69). Independent assessment demonstrated more alterations of body representation in participants with FM compared to controls: out of the 20 participants with FM and the 24 pain-free participants, 18 (90.0%) participants with FM and 10 (41.7%) controls displayed distortions in at least 1 of the 3 Body regions. Twelve (60.0%) participants with FM and 5 (20.8%) pain-free controls showed alterations in all 3 Body regions. The trunk was the site with the most alterations for both groups (80.0% of FM participants and 33.3% of controls reported distortions), while the Lower limb and the UL were the least affected in the FM group (52.5%) and the CTRL group (14.6%), respectively. Body representation anomalies were multiform and were notably represented as changes in size (enlargement or shrinkage), shape, texture (hardness, sharpness, etc.), weight or pressure, as an absence or an imprecision regarding the delimitations of certain body parts (blurry, discontinued, etc.), and as the impression of having an external painful stimulus on/in the body (knife, needle, fire, etc.). To summarize, the body drawings of the FM group showed clear, frequent, and multiform distortions, whereas the body drawings of the CTRL group presented little to no anomalies.

## 4 Discussion

The main goal of this study was to compare the TDE and the body representation of individuals with FM and pain-free individuals. Results show no difference in TDE between the groups, across the three Body regions (UL, LL, trunk). This result contradicts our hypothesis, which states that pain in participants with FM would perturb TDE. In the group with FM, we especially expected to observe more significant TDE errors at the trunk, where pain intensity was higher than in the upper and lower limbs, but this was not the case. This observation contrasts with the analysis of the body drawings, which exhibited clear and frequent distortions of the body image in the group with FM compared to the CTRL group, especially at the trunk level. The results of the TDE task will be discussed first in terms of intergroup comparison and then in relation to the distribution of pain in participants with FM. Then, the body drawings will be interpreted and contrasted with the TDE results. Lastly, some limitations will be outlined.

The examination of the signed TDE shows an increase in the estimations according to the interstimuli distances for both groups. This result confirms that participants did not estimate randomly and could discriminate between the three interstimuli distances. TDE errors were not statistically different between the groups and across Body regions. This is consistent with a study that showed no difference of TDE in participants with CRPS and in a non-CRPS UL chronic pain group, compared to pain-free participants, on the painful oron-painful hand (38). No study has investigated TDE in individuals with FM. However, other tactile perception assessments, such as the two-point discrimination threshold (TPD) have shown contradictory results. This measure represents the interstimuli distance at which the participants feel two tactile stimuli, as opposed to one tactile stimulus. Recently, Menten and colleagues reported no alterations of TPD in participants with FM at the cervical level, the lumbar level, on the hands, and the feet (20). In contrast, Martinez et al. showed higher TPD thresholds in the FM group at the cervical level (28). The strength of the present study was in providing a global assessment of TDE, with measurements over three Body regions, each stimulated on two different Body sites. This result generated a more representative depiction of tactile perception in individuals with FM, which showed no alterations.

The group with FM felt more intense pain on the trunk than on the UL and LL, but contrary to our hypothesis, TDE errors were not larger in this region. This observation is in contradiction with studies showing a link between tactile perception alterations (as measured with the TDE or the TPD) and clinical variables such as more intense pain, longer duration of pain, and a higher disability score in patients with non-FM chronic pain (37, 62–64). However, in our study, pain intensity differences between more painful and less painful Body regions (on the day of the study) may not have been as pronounced as in other studies since all three regions were considered painful at the group level (the VAS ranged from a median of 2.25 for the UL to a median of 4.75 for the trunk). FM is a widespread chronic pain syndrome, meaning that pain is felt on all four quadrants of the body (39). Studies show that individuals with FM report pain in multiple body regions, with about half of them indicating feeling pain “all over the body” (65, 66). This observation can limit the comparison of “painful” and “not painful” regions.

Another factor which may have played a part in the TDE results is the potentially different reference frames involved in the task. While applying the tactile stimuli, participants had their eyes closed and could not see the caliper. This may have promoted the use of an egocentric reference frame, the body-centred reference frame important for action which determines the relationship between the self and the world (67). The participants then opened their eyes to estimate the interstimuli distance with their fingers on the tablet. This, on the other hand, might have encouraged the use of an allocentric reference frame, the world-centered reference frame crucial for perception which establishes the connection between external objects (such as the points of the touchpad pressed by the fingers and the boundaries of the touchpad) (67). The use of an egocentric reference frame is promoted by the availability of information about the body, such as tactile or proprioceptive sensations, whereas the adoption of an allocentric reference frame is encouraged by the presence of information from the external environment (allocentric information), such as the vision of objects or of other people (68). In a study that aimed to dissociate the factors influencing the use of the two reference frames, participants either had to throw a bag at the end of a line painted on the ground in front of them (motor task) or verbally estimate the length of the line (perceptual task). A visual illusion was used to give the impression that the line was shorter or longer than it was. The researchers hypothesized that participants’ performance would be impacted by the visual illusion in the perceptual task because they would rely on allocentric visual information, which was verified. On the other hand, they supposed that performance would not be perturbed in the motor task because participants would use egocentric information (such as the proprioceptive and visual information of the throwing arm) to estimate the distance at which they needed to throw the bag to reach the end of the line***.*** This hypothesis was only partially confirmed: when participants were placed close to the line, the illusion had no effect on performance, but when they were 1.5 meters away from the line, the visual illusion influenced throw length. According to the authors, in this last condition, the additional allocentric information may have rendered the egocentric information less potent, thus promoting an allocentric reference frame (68). In our task, the additional allocentric visual information available when participants opened their eyes to estimate the interstimuli distance might have encouraged a switch from an egocentric to an allocentric reference frame, which could have influenced the TDE (68, 69).

A similar paradigm as ours was used in a TDE study involving participants with chronic pain. In this study, participants with chronic low back pain were stimulated on the lower back. They had to estimate the interstimuli distance by showing the distance on a non-graduated caliper with their eyes open. This test showed excellent intra-examiner reliability and moderate to excellent intra-examiner reliability. Moreover, pain characteristics such as distribution, duration, and pain intensity explained 42% of the TDE variance observed in participants with chronic pain (37). To summarize, although a switch in the reference frame was used to feel the interstimuli distance and estimate it could have influenced our results, a study on chronic pain participants showed promising results using a similar paradigm.

The body drawings demonstrated evident felt distortions of body image in participants with FM, compared to pain-free controls. The trunk, the most painful region in participants with FM, was the most altered region, though this was true for controls, also. A few other studies have used body drawings in chronic pain populations and have demonstrated body image alterations at painful sites, such as changes in the shape and the size of body parts and “missing” (i.e., not felt) body parts (20, 36, 70).

To summarize, compared to controls, we expected to observe tactile perception alterations (as expressed by larger TDE errors) and body representation perturbations (as indicated by distorted body drawings) in participants with FM. The hypothesis was that alterations in tactile perception would perturb the multisensory integration of tactile information with other sensory information (such as visual information) and thus impact body representation. However, a contrast was observed between the absence of difference in TDE errors between groups and the differences in body image. Two recent studies have reported analogous contrasts between an unaltered tactile perception and a distorted body image in chronic pain patients (20, 38). Participants with CRPS perceived both their painful and non-painful hands as bigger than they were but displayed no difference in TDE, compared to pain-free controls (38). In participants with FM, body drawings revealed distortions, whereas tactile perception on the cervical and lumbar regions was not different from controls (20). Similarly, studies using sensory training to influence sensory perception showed that visual training (i.e., being exposed to expanded or contracted images of a body part) influenced body image but not tactile perception (71). On the contrary, tactile training (i.e., repeated pairs of tactile stimulation on the hand, with an interstimuli distance shorter or larger than a test interstimuli distance) did not influence hand size representation despite impacting TDE (72). This observation suggests that factors other than tactile information may be associated with the body representation alterations observed at a conscious level. These factors could include the relative reliance on visual information (9, 73–76) and prior knowledge about the body, such as acquired knowledge about the size and shape of our body parts (77, 78).

The dissociation between distorted body drawings and unaltered TDE can also be interpreted in terms of body image and body schema. Body drawings are used as a measure of body image (20, 36, 70), whereas the TDE can be interpreted as an assessment of the body schema, as it is an unconscious touch-based estimation of the length of a body part (7, 28, 79). In this regard, the present findings could indicate body image alterations in individuals with FM, with a preserved body schema. These two types of body representations, although not entirely independent (80, 81) have historically been separated (1). Examples of dissociation between body image and body schema include deafferented patients (i.e., a loss of tactile and proprioceptive information), who show impairments of the body schema with intact body image (1) and patients with numbsense [i.e., “a tactile deficit with preserved tactually guided movements” (81)], who present a disrupted body image with no alterations of the body schema (3). Our findings are consistent with a dissociation between these two body representations in individuals with FM.

Some limitations can be outlined. First, the IPAQ indicated that our sample of participants with FM may be more physically active than has been reported in other studies: the continuous MET score indicated a median of 3,440 MET-min/week compared to a median of 495 (82), 2,261 (83), and 2,741 MET-min/week (84). This result could suggest that the sample was generally less severely affected and/or impacted by symptoms as other individuals with FM. However, the apparent distortions of body perception and the range of pain intensity hint at unequivocal alterations, making this explanation less likely.

To adapt to differences in sensitivity across Body sites and participants, the pressure of the caliper was adjusted according to the participants’ feedback. The goal was for the tips of the caliper to be felt without being painful. This non-standardized application may have influenced the results. Indeed, a recent study showed that more intense pairs of tactile stimuli were perceived as farther apart than less intense pairs of tactile stimuli (85). A pressure sensor integrated into the caliper could have provided additional information about the applied pressure, potentially relevant to interpreting our findings.

Finally, body drawings have been used in several studies involving individuals with chronic pain (20, 36, 86), but no standardised and validated method has been developed yet. The qualitative assessment of the drawings by two independent evaluators shows more frequent and obvious distortions in the group with FM, compared to the CTRL group. Since this evaluation is subjective, all the drawings are provided in the Supplementary Material for readers to determine if they agree on this conclusion or not.

In conclusion, no difference in TDE errors was found between participants with FM and CTRL participants. Still, clear and frequent alterations of the body image were observed in individuals with FM. This suggests a preserved integration of tactile information but an altered multisensory integration of this information to generate a precise and accurate body representation at a conscious level. It could also indicate a dissociation between an intact body schema and a perturbed body image in FM. Future research should investigate the relative contribution of tactile and visual information and prior knowledge about the body in the generation of body representation in individuals with FM to determine the role of each information in the observed perturbations. Clinical research could also be carried to develop therapies aimed at improving body image. For example, virtual reality could help normalise body image in clinical populations (87, 88). Moreover, given the fact that 90% of individuals with FM reported alterations in body image, it would be worthwhile to consider integrating information in relation to that in educational programs targeting this population.

## Data Availability

The raw data supporting the conclusions of this article will be made available by the authors, without undue reservation.
