# Supplementary figures and images for: Preserved tactile distance estimation despite body representation distortions in individuals with fibromyalgia
Source: Front Pain Res (Lausanne). 2024 Jul 25;5:1414927. doi: 10.3389/fpain.2024.1414927 (PMC11306202; doi:10.3389/fpain.2024.1414927)

## Slide 1
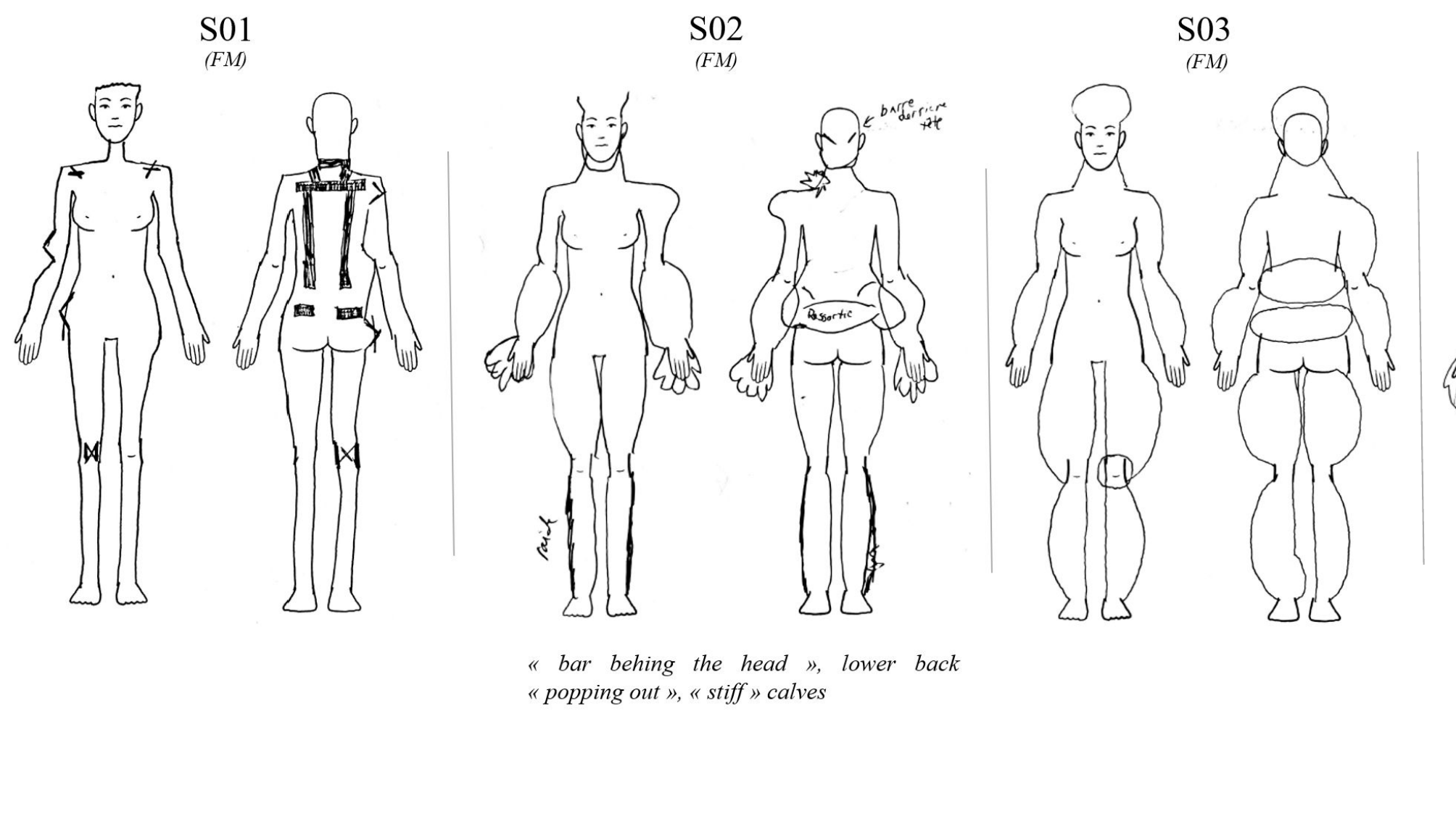

Supplement: Supplementary file 1 [file Presentation1.pptx]
